# Notes of the Gettysburg Anniversary

**Published:** 1913-08

**Authors:** 


### Notes of the Gettysburg Anniversary

Probably there have never been gathered together so many old men as on this occasion. Accurate statistics are not available, but about 50,000 veterans, including five per cent. or less of Confederates were assembled, most of them being accommodated in tents, served with rations and given the benefits of the most modern camp hygiene. Probably half as many other visitors were present. Order was maintained by the State Constabulary, details of soldiers, regulars and national guard and much help was given by boy scouts. Dispensary and hospital services were maintained by the State Department of Health, the Red Cross Society and the Medico-chirurgical Hospital of Philadelphia, which sent an automobile ambulance and a corps of surgeons and nurses. Perhaps on account of the divided responsibility we were unable to learn the exact number of deaths and cases treated. The estimates of the former ranged from six by one of the surgeons of the State Health Department to seventeen by the veterans, based on the assumption that deaths were announced by the number of guns fired at reveille. Later reports state the number of deaths at nine. At least 250 cases of "heat prostration" were treated, but the somewhat uncharitable view was expressed that the heat was measured more appropriately by a column of alcohol than of mercury. As the youngest veteran must have been close to 65, and as there were many men in the 80's, a few in the 90's, including the sole surviving commanding general, Daniel E. Sickles; and one centenarian, Micajah Weiss, aged 110, the maximum estimate of deaths is surprisingly low. The average (census) mortality for the ages 65-74 is 58.3 :1000 for those above 75, 142 :1000. We should, therefore, expect an average weekly mortality of 50—100 in such a gathering and the fatigue of travel, excitement, exposure to heat, temptation to various excesses, etc., should compensate for any ordinary seasonal fluctuation. Two of the deaths of veterans occurred in a runaway accident, otherwise all the deaths of which we could learn were of the ordinary types of sudden death in the aged. None of the casualties or medical cases were of professional interest, although four or five men were stabbed as the result of a purely figurative insult to the memory of the late President Lincoln's mother. This occurred in the village and did not involve the veterans at all.

Considering the addition of approximately 75,000 persons to a village of about 3500, there was surprisingly little suffering for food and lodging except by the fastidious and comparatively little extortion, although, in view of the small means of many of the veterans, one might have asked for some provision which would have spared their feeble steps.

No attempt was made at statistics of the number of veterans who had participated in the battle. Apparently, most of the Confederates had and, judging by impressions from many groups, a third of the Union veterans. At first thought, this seems impossible, but the following facts lend probability to the rough estimate. The average mortality of all the Union forces was 10.5 per cent. 95,358 Union soldiers participated in the three day's fight and about 85,000 would have survived the war. According to Homan's table of survivors (acknowledged to be low for selected insurance risks but probably fairly accurate for a general population for the period covered) about 25,000 would now survive if the average age had been 25 in 1865; 14,000 if the average age had been 30. It is fair to assume that there are about 20,000 survivors at present.

While Gettysburg was the bloodiest fight known to modern military history, the following official statistics are almost disappointingly small in direct mortality:

| | Federal. | Confederate. |
|---|---|---|
| Men Engaged | 95,358 | 75,256 |
| Killed | 3,155 | 2,592 |
| Wounded | 14,529 | 12,705 |
| Missing or Captured | 5,365 | 5,150 |
| Total Losses | 23,049 | 20,447 |
| Percent of Loss | 24.1 | 27.1 |
| Percent of Killed and Wounded | 18.5 | 20.3 |

We still speak of "decimated" ranks, but the reduction of even a single unit to a tenth of its original force is of rare occurrence since the days of hand to hand conflict. We noted one regiment, already much depleted, which was reduced from 220 to 37 effective men. The charges of the Louisiana Tigers and of Pickett's men resulted in an aggregate loss (killed, wounded and missing) of about two-thirds.

Several interesting, though gruesome points, were developed in conversation regarding the significance of the term "missing." While, in long campaigns or exhausting marches, the word includes a considerable number of deserters and stragglers, all the veterans agreed that, for a battle, the "missing" were either prisoners or unidentified dead and it was stated that the number of unidentified dead in national cemeteries warranted the general statement that the official figures of "killed" should, on the average, be increased by approximately 50 per cent. The reasons for lack of identification emphasized one of the horrors of war scarcely comprehensible by one accustomed to death only as it occurs in times of peace. We know academically that rigor mortis occurs with greater rapidity when the muscles are surcharged with products of catabolism from active exercise, though we sometimes doubt the accounts of dead soldiers stiffened at the moment of death so as to retain the posture in which they happened to be. Yet so many such accounts are given regarding this and other battles that our skepticism is perhaps not justified. We also know academically that the sooner rigor mortis sets in, the sooner it terminates in decomposition and that decomposition is retarded by even incomplete embalming, protection from the sun's rays and heat. Yet it was a surprise to hear veterans speak, as a matter of course, of the bodies of the Louisiana Tigers being "black and bloated" the day after the charge. Most of the deaths in the "Wheat Field" occurred on the afternoon of July 2 and on the following day. Before Lee's retreat on the 4th was an assured fact to the Union troops, burial parties were sent out, in the evening, because of the terrible odor at a distance of half a mile or more, when there was considerable danger that flags of truce could not be seen by the enemy. It can readily be imagined, that under such circumstances, not only the disfigurement but the disinclination to search closely for marks of identification and the necessity of haste would result in a relatively large proportion of "unknown dead," even among acquaintances of the burial party, while very few graves of the enemy would be marked. It was generally stated that decomposing human bodies were much more offensive than those of animals, horses especially sharing with men, the risk of battle. Lack of identification was also ascribed to the custom of stripping the bodies of both friend and foe, by the Confederates, and this custom was defended as necessary on account of the pitifully meagre equipment of many of the Confederate troops. One of the burial party in the Wheat Field had also served similarly about ten days after the second battle of Bull Run. The first body that they attempted to remove to a trench burst and spread over several feet so that they learned to cover bodies as they lay by merely piling earth over them from both sides

The rocky nature of Little Round Top and the adjacent hills suggested the presence of rattle snakes. It occurred to us that, in such regions and in the swamps of the Peninsular campaign, snake bites might have caused many deaths. To our surprise all the veterans interrogated said that they had never even heard of such a case and all but one said that they did not remember seeing a snake during any massed movements of troops, the exception being a badly frightened black snake that ran through a camp. This suggested that the noise or odor of large bodies of men drive snakes from the vicinity.

Near the equestrian statue of General Hancock we encountered two veterans who had been near him at the time he was wounded. During his presidential candidacy some of his opponents were low enough to refer to him as emasculated, although, if true, his mutilation, received in action, would have been as honorable as the loss of a limb. The facts, as stated, are that he was wounded while on horseback directing the battle; either directly by a bullet or by a saddle nail driven by the bullet, producing a painful wound of the soft parts between the testicles.

A commendable feature of the great gathering was, so far as could be judged, an entire absence of the female birds of prey that are usually so conspicuous. We did not observe a single instance of impropriety of even the mildest degree nor see a person who did not look respectable.

Attendance at the various exercises in what was said to be the largest tent in the world, impressed us with the strangeness of discourses which were not highly technical. Perhaps, as a profession, we neglect lectures and discourses that are neither medical nor closely analogous to medical papers in their technicality. The speakers, military, political and clerical—including one gentlemen who spoke the most peculiar high-class Anglo-American dialect that we have ever heard—all rightly emphasized the importance of the battle, but went rather too far in ascribing to it the virtual settlement of the Rebellion. It is true that it concluded the attempt of the south to invade the north and that it was a great northern victory, but the battle itself can scarcely be considered as a defeat of the southern cause. As many veterans expressed it, in partial justification of the failure to follow up the victory, the Union commanders did not know till a day or two after the battle that they really had defeated Lee, and this is only another way of saying that, in spite of the superiority of numbers and advantages of being on the defensive for the first time in any great battle of the war, the battle itself was not the decided victory which it is usually considered. Lee was already withdrawing toward the north when the encounter at Gettysburg took place; while repulsed in his offensive movements his losses were absolutely smaller and relatively only slightly greater than his opponents, and his continued retreat was due to the necessity of reaching a base of supplies. It required a year and three-quarters of hard fighting to give to the battle of Gettysburg its significance as a turn of the tide. We are inclined to believe that there are very few crises in any line of human effort, which do not require continued hard fighting to give them their critical value.

